# Supplementary figures and images for: A BODIPY based probe for the reversible “turn on” detection of Au(III) ions
Source: Turk J Chem. 2021 Dec 18;46(2):523–9. doi: 10.3906/kim-2110-22 (PMC10734706; doi:10.3906/kim-2110-22)

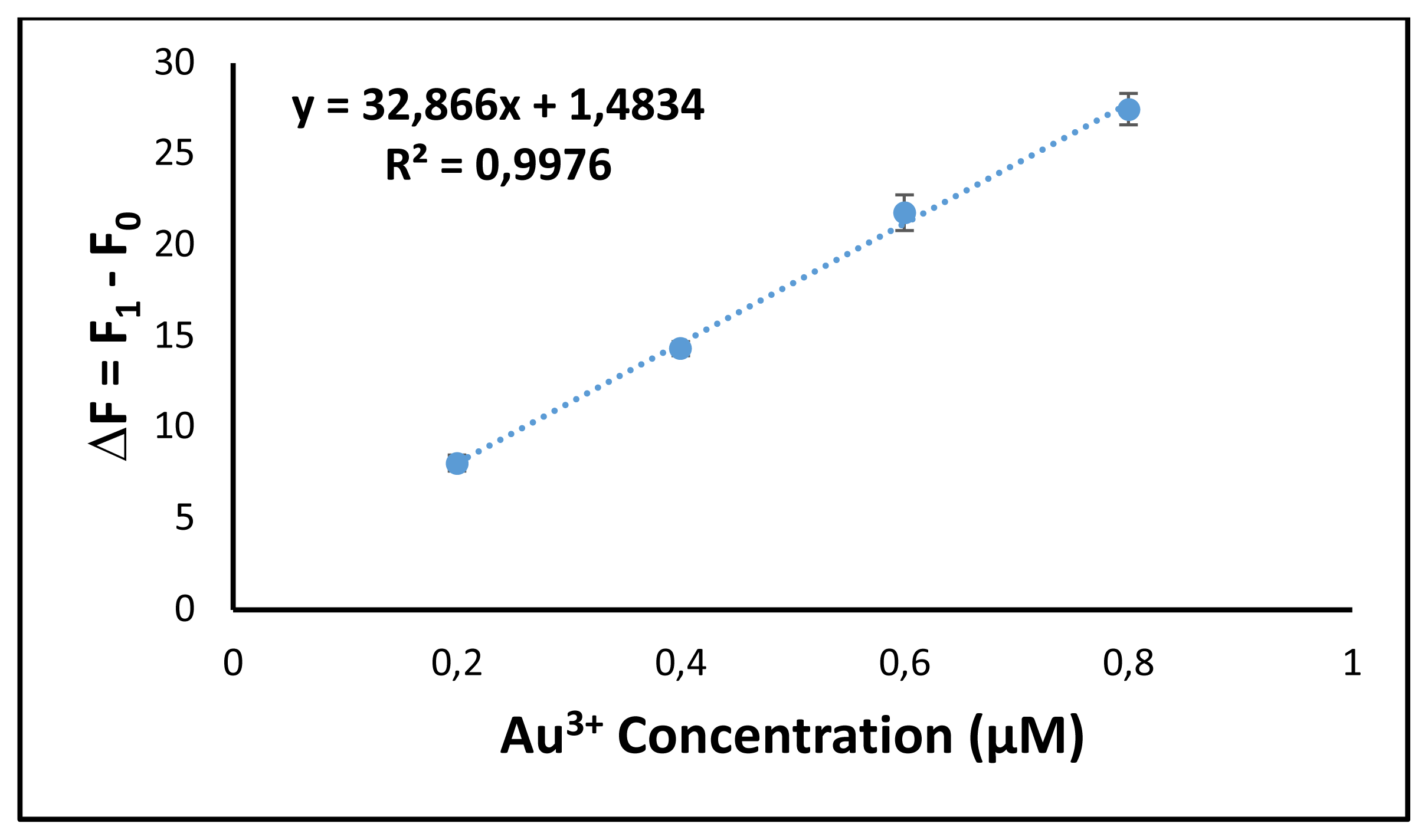

Supplement: Figure S1 — Fluorescence changes of BOD-AP (10 μM) upon addition of Au3+ (0.2 to 0.8 μM, 0.02 to 0.08 equiv.) in 0.1 M phosphate buffer, pH 7.0/EtOH (v/v, 1:1) (λex = 460 nm, λem = 511 at 25 °C). [file turkjchem-46-2-523s1.tif]

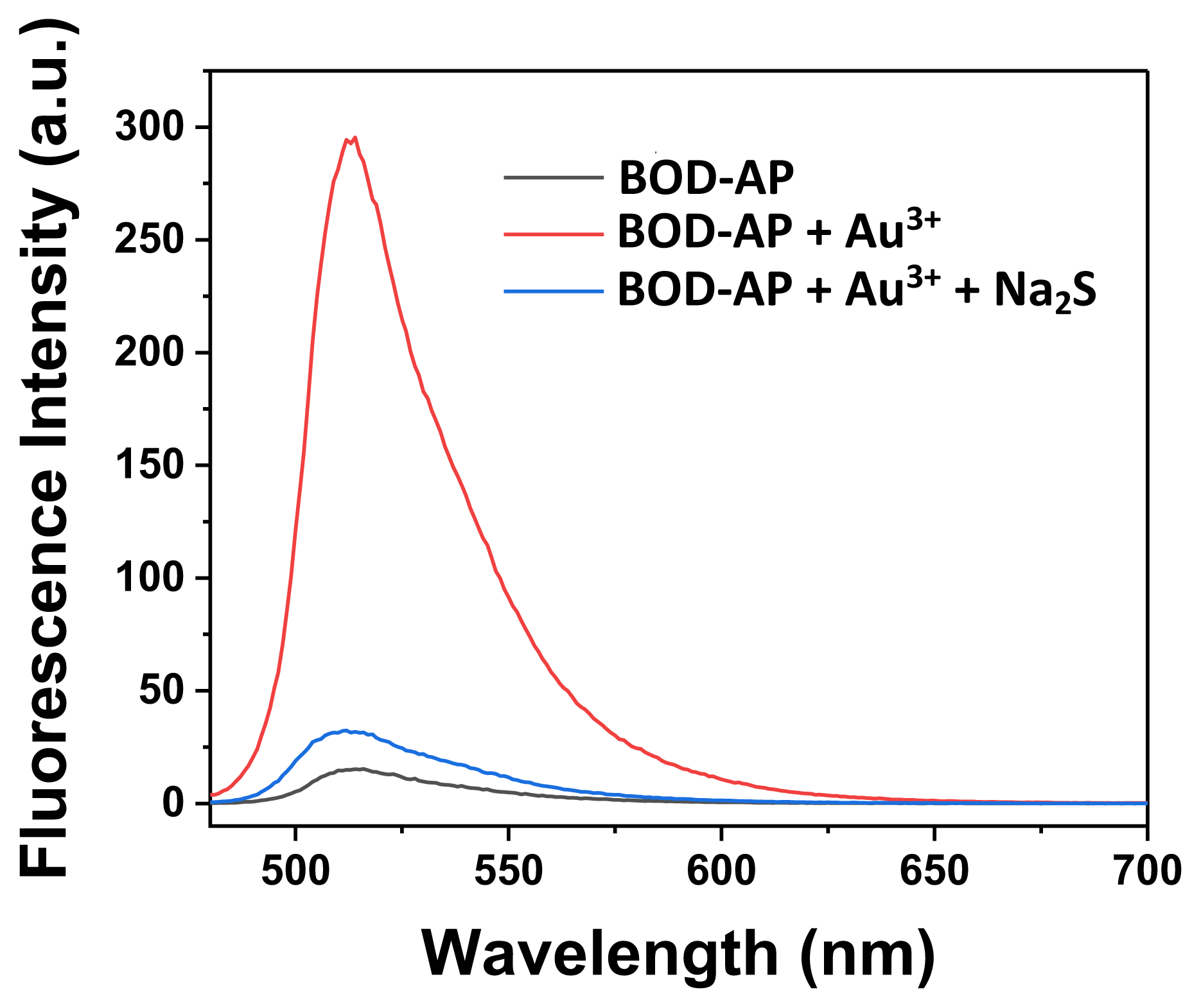

Supplement: Figure S2 — Fluorescence spectra of BOD-AP (10 μM) (black), BOD-AP (10 μM) + Au3+ (20 μM, 2 equiv.) (red), BOD-AP (10 μM) + Au3+ (20 μM, 2 equiv.) + Na2S (20 μM, 2 equiv.) (blue) in 0.1 M phosphate buffer, pH 7.0/EtOH (v/v, 1:1) (λex = 460 nm, at 25 °C). [file turkjchem-46-2-523s2.tif]

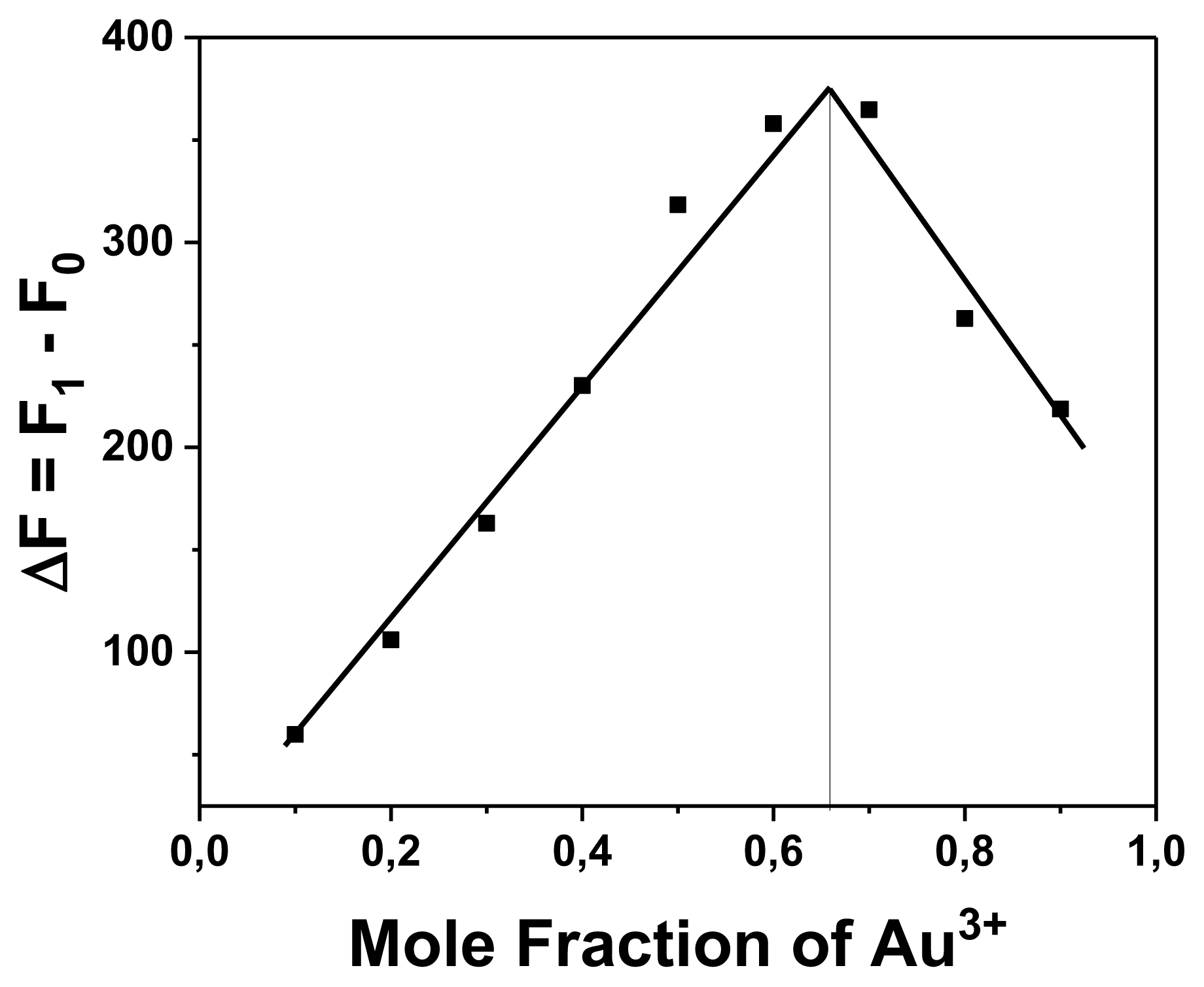

Supplement: Figure S3 — The Job’s plot analysis between BOD-AP and Au3+ in 0.1 M phosphate buffer/EtOH (pH 7.0, v/v, 1:1) The total concentration of BOD-AP and Au3+ was kept constant at 20 μM (λexc = 460 nm, λem = 511 nm at 25 °C). [file turkjchem-46-2-523s3.tif]

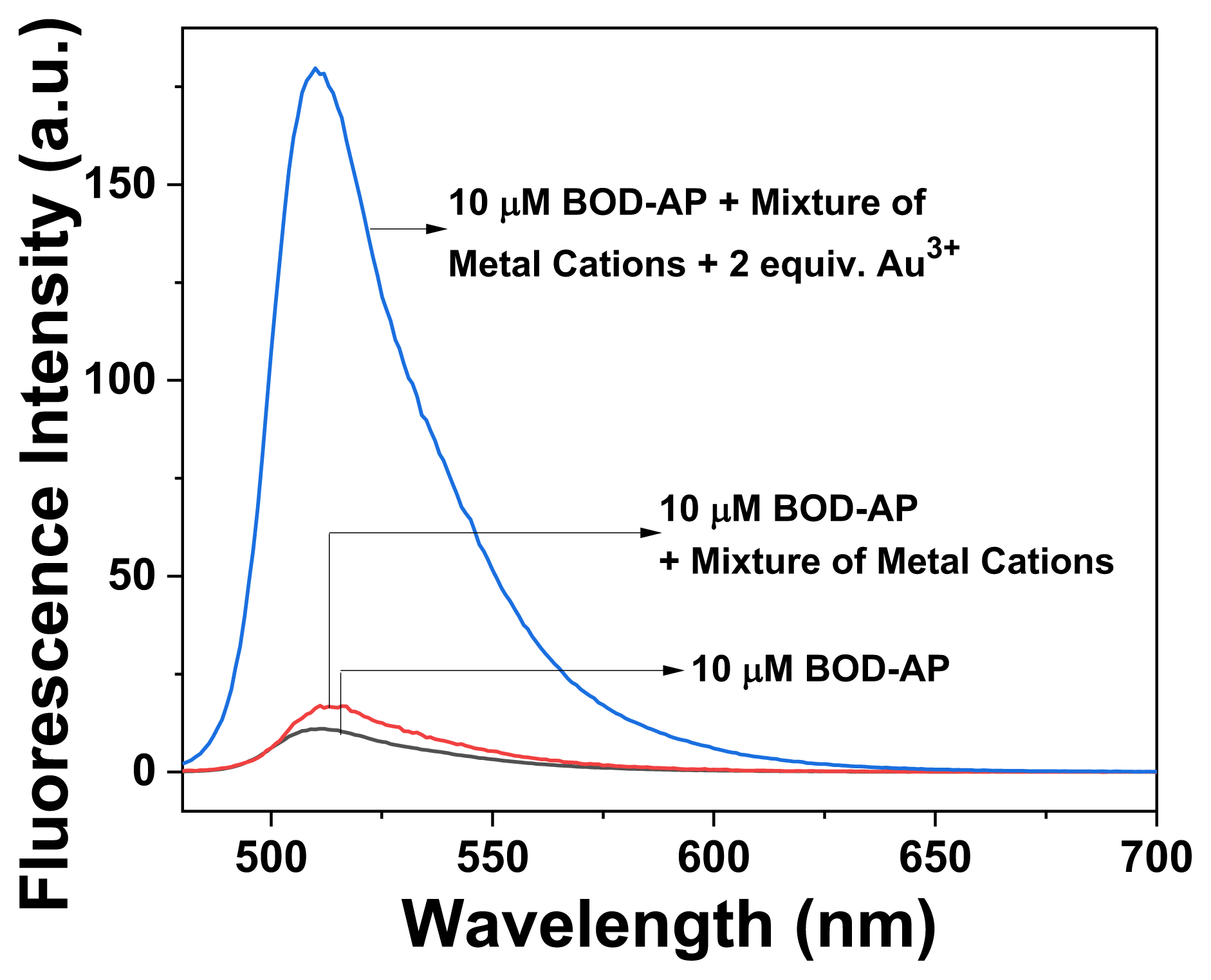

Supplement: Figure S4 — Fluorescence spectra of BOD-AP (10 μM) (black), BOD-AP (10 μM) + mixture of metal cations (including Na+, K+, Li+, Ca2+, Mg2+, Ba2+, Ag+, Hg2+, Zn2+, Pb2+, Ni2+, Cd2+, Fe2+, Cr3+, Ce3+, and Al3+) (100 μM, 10 equiv.) (red), BOD-AP (10 μM) + mixture of metal cations (100 μM, 10 equiv.) + Au3+ (20 μM, 2 equiv.) (blue) in 0.1 M phosphate buffer, pH 7.0/EtOH (v/v, 1:1) (λex = 460 nm, at 25 °C). [file turkjchem-46-2-523s4.tif]

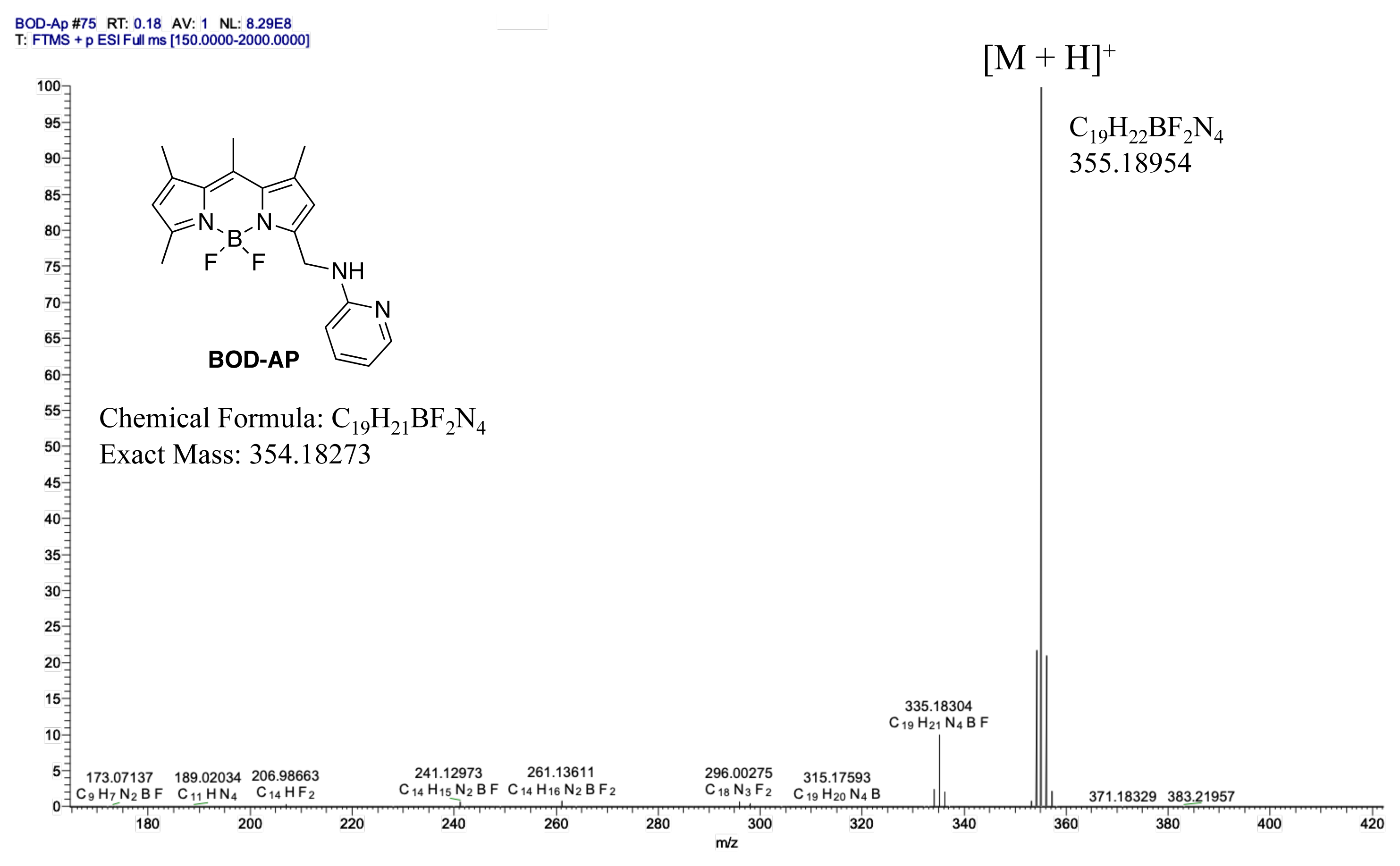

Supplement: Figure S5 — HRMS Spectrum of BOD-AP. [file turkjchem-46-2-523s5.tif]

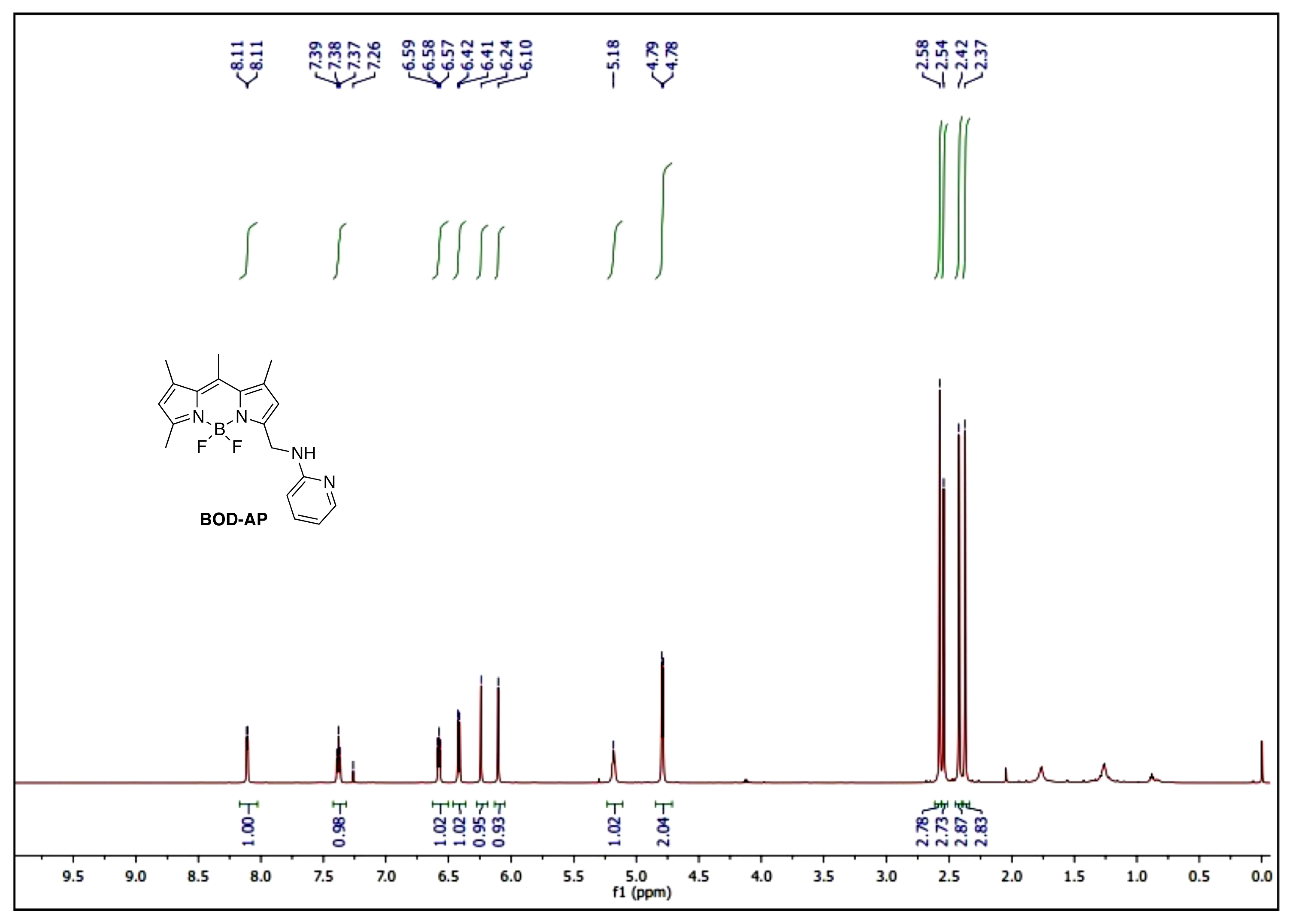

Supplement: Supplementary file 6 [file turkjchem-46-2-523s6.tif]

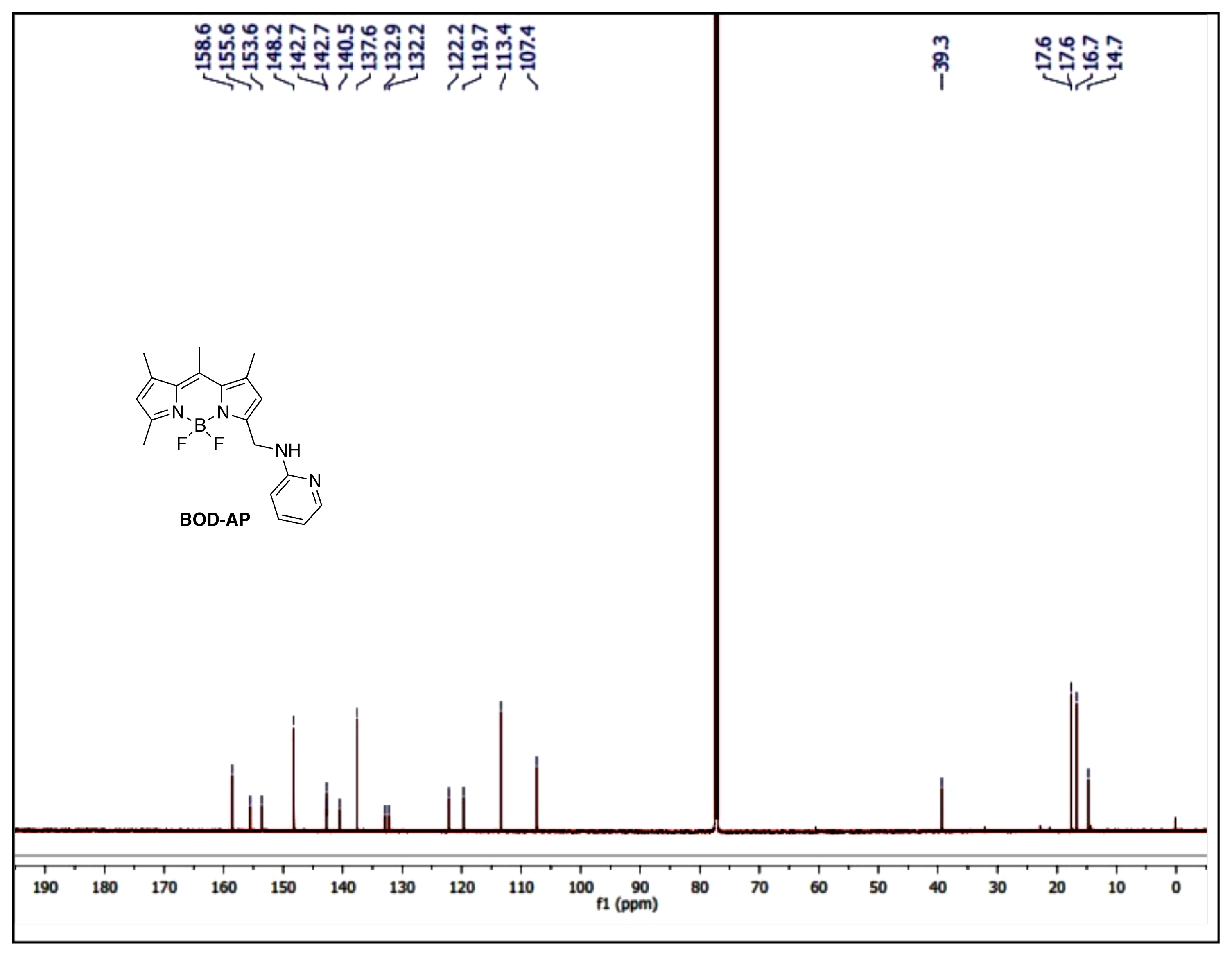

Supplement: Supplementary file 7 [file turkjchem-46-2-523s7.tif]
